# Integrative Bioinformatics Analysis Reveals CHEK1 and UBE2C as Luminal A Breast Cancer Subtype Biomarkers

**DOI:** 10.3389/fgene.2022.944259

**Published:** 2022-07-12

**Authors:** Daowu Yu, Shengwei Liu, Yijun Chen, Lumeng Yang

**Affiliations:** Yongchuan Hospital of Chongqing Medical University, Chongqing, China

**Keywords:** breast cancer, WGCNA, CHEK1, UBE2C, timer

## Abstract

In light of the limited number of targetable oncogenic drivers in breast cancer (BRCA), it is important to identify effective and druggable gene targets for the treatment of this devastating disease. Herein, the GSE102484 dataset containing expression profiling data from 683 BRCA patients was re-analyzed using weighted gene co-expression network analysis (WGCNA). The yellow module with the highest correlation to BRCA progression was screened out, followed by functional enrichment analysis and establishment of a protein–protein interaction (PPI) network. After further validation through survival analysis and expression evaluation, CHEK1 and UBE2C were finally identified as hub genes related to the progression of BRCA, especially the luminal A breast cancer subtype. Notably, both hub genes were found to be dysregulated in multiple types of immune cells and closely correlated with tumor infiltration, as revealed by Tumor Immune Estimation Resource (TIMER) along with other bioinformatic tools. Construction of transcription factors (TF)-hub gene network further confirmed the existence of 11 TFs which could regulate both hub genes simultaneously. Our present study may facilitate the invention of targeted therapeutic drugs and provide novel insights into the understanding of the mechanism beneath the progression of BRCA.

## Introduction

As the most aggressive malignancy in females, breast cancer (BRCA) affects approximately one of every nine women globally and is the leading cause of cancer-associated mortality worldwide (Bray et al., 2018). Despite the huge advancements of BRCA screening and surgical techniques during recent decades, over 20% of BRCA patients still develop recurrence and finally result in dismal outcomes (Early Breast Cancer Trialists' Collaborative Group, 2015; Waks and Winer, 2019). Therefore, exploring novel biomarkers underneath BRCA progression and deciphering their underlying mechanisms will facilitate the development of therapeutic drugs and promote survival rate of BRCA patients.

As one of the most frequently applied bioinformatics algorithms to discover cancer-related biomarkers and signaling pathways, the weighted gene co-expression network analysis (WGCNA) approach provides a systematic strategy for in-depth mining of phenotypic features of interest (Langfelder and Horvath, 2008). Compared with traditional analytical methods that mainly focus on individual genes, WGCNA converts gene expression data into co-expression modules and highlights the correlation between a specific gene network and the phenotype of cancer. In addition, WGCNA implements methods for both weighted and unweighted correlation networks and provides a more effective mean to explore the hub genes that regulate critical biological processes (Barabási et al., 2011). To date, WGCNA has been successfully applied for identifying potential biomarkers with diagnostic and prognostic values in a wide range of cancer types (Liu et al., 2021; Ma and Li, 2021; Yang et al., 2021).

Tumor Immune Estimation Resource (TIMER) is an *in silico* deconvolution method to estimate tumor immune infiltration (Li et al., 2017; Li et al., 2020). Through integration of six state-of-the-art algorithms, this method can explore the associations between immune infiltrates and genetic or clinical features from expression profiles of tumor. Accumulating evidences suggest that this emerging tool provides comprehensive analysis for the identification of signatures in tumor-infiltrating immune cells and prediction of tumor-immune interactions (Chen et al., 2020; Kim et al., 2020; Zhang et al., 2021).

In the present study, WGCNA was constructed by using the GSE102484 dataset containing gene expression profiling data from 683 BRCA patients. Specific module associated with BRCA progression and metastasis was identified, followed by hub gene prediction and functional analyses. Notably, TIMER algorithm was further applied to unravel the relationship between hub gene expression and tumor immunity. The regulatory network of the identified hub genes was also explored.

## Materials and Methods

### Data Collection

Human BRCA dataset GSE102484 with patient clinical information was downloaded from GEO database (Cheng et al., 2017). GSE102484 dataset was performed on platform GPL570 Affymetrix Human Genome U133 Plus 2.0 Array (HG-U133_Plus_2). GSE102484 includes 683 BRCA samples, which were used to construct a co-expression network, followed by distinguishment and extraction of the hub genes. R package was used to annotate raw data, generate an expression matrix, and match probes with official gene symbols. The median absolute deviations (MADs) were ranked from largest to smallest, and the expressions of the top 25% genes with the largest differences in the samples were extracted for in-depth analysis.

### Weighted Gene Co-Expression Network Analysis

The R package “WGCNA” was utilized to perform weighted gene co-expression network analysis on selected genes to find out the expression patterns between different genes. Genes with similar expression patterns were grouped into a specific module and marked with a unique color. Then, the correlation between these modules was calculated and a heat map was depicted to show the independence between each module. Next, the correlation analysis was conducted to find modules related to the chosen clinical information. Module with the most significant correlation to tumor progression was selected for further analysis.

### Gene Ontology and Kyoto Encyclopedia of Genes and Genomes Pathway Enrichment Analyses

To investigate the biological functions and signal pathways involved in genes of the yellow module, the group of genes in the yellow module were analyzed through the R package “clusterProfiler” (Yu et al., 2012). Then, another R package “ggplot2” was applied to screen out the top 10 significantly representative terms of Gene Ontology (GO) and Kyoto Encyclopedia of Genes and Genomes (KEGG) pathway analyses. Enriched GO terms and KEGG pathways were identified based on the cut-off criterion of *p* < 0.05.

### Protein–Protein Interaction of the Key Module Genes

For the purpose of hub genes selection, genes of the yellow module were uploaded to the STRING database to build a protein-protein interaction (PPI) network (Szklarczyk et al., 2019). The minimum interaction score > 0.4 was set up as the threshold of the key genes in the PPI network. Then, a Cytoscape plug-in cytohubba was used to extract the top 10% targets in the yellow module based on the degree method (Shannon et al., 2003).

#### Identification and Validation of Hub Genes

According to the results from the cytohubba analysis, the top 56 genes in the yellow module were selected as hub gene candidates for further analysis. The GEPIA webserver (Tang et al., 2017) (http://gepia.cancer-pku.cn/) was next used to perform the overall survival and mRNA expression analyses of the candidates. Hub gene candidates with the significant results were selected for the next round of analysis. Successively, the Kaplan–Meier plotter (http://kmplot.com/analysis/) was used to draw the survival plots to verify the outcomes so as to screen out the real hub genes. To be specific, the PAM50 module was selected to classify the BCRA subtypes, while the others were set as default. Subsequently, GEPIA 2021 (Li et al., 2021) (http://gepia2021.cancer-pku.cn/), a standalone extension with multiple deconvolution-based analysis for GEPIA, was used to visualize the gene expression in each cell type selected with the interactive boxplot and perform the cell type-level differential expression analysis of the identified key targets comparing with normal breast tissues. Therefore, we can determine the transcription level of hub genes at certain types of cells in BRCA tissues. *p* < 0.01 was considered to be statistically significant. Moreover, TIMER2.0 (Li et al., 2020) (http://timer.comp-genomics.org/), a comprehensive resource for systematical analysis of immune infiltrates across diverse cancer types, was used to present the co-expression analysis of real hub genes in the BRCA subtype.

### Immune Infiltration Level Analysis

Based on the findings above, the immune association module of TIMER2.0 was then applied to explore the correlations between expression of hub genes and immune infiltration level in luminal-A subtype of BRCA. Tumor purity was used as a major confounding factor in this analysis. Since the majority of immune cell types are negatively correlated with tumor purity, we selected the “Purity Adjustment” option which used the partial Spearman’s correlation to perform the association analysis.

#### Immune Correlation and CARE Score Analyses

The crosstalk between tumor and immune system plays an essential role in cancer initiation, progression and treatment. Thus, TISIDB(Ru et al., 2019), an integrated repository portal for tumor-immune system interactions, was used to explore the relations between three types of immunomodulators (immunoinhibitors, immunostimulators and MHC molecules) or chemokines and expression of hub genes. The Computational Analysis of REsistance (CARE) software (Jiang et al., 2018) was further used to identify genome-scale biomarkers of targeted therapy response using compound screen data. It could search for CARE results on three datasets of CCLE, CTRP, and CTRP. For each gene, the CARE score indicates the association between its molecular alteration and drug efficacy. A positive score indicates a higher expression value (or presence of mutation) to be associated with drug response, whereas a negative score indicates drug resistance.

#### Construction of Transcription Factors-Hub Genes Network

In order to identify the transcription factors (TFs) that modulate hub gene expression and reveal their regulatory relationships, a regulating network on account of hub genes and TFs in BRCA was constructed with the help of Cytoscape software. Then, the plugin iRegulon of Cytoscape was applied to forecast TF regulation networks.

## Results

### Construction of the Co-Expression Network and Identification of Modules

**FIGURE 1:**
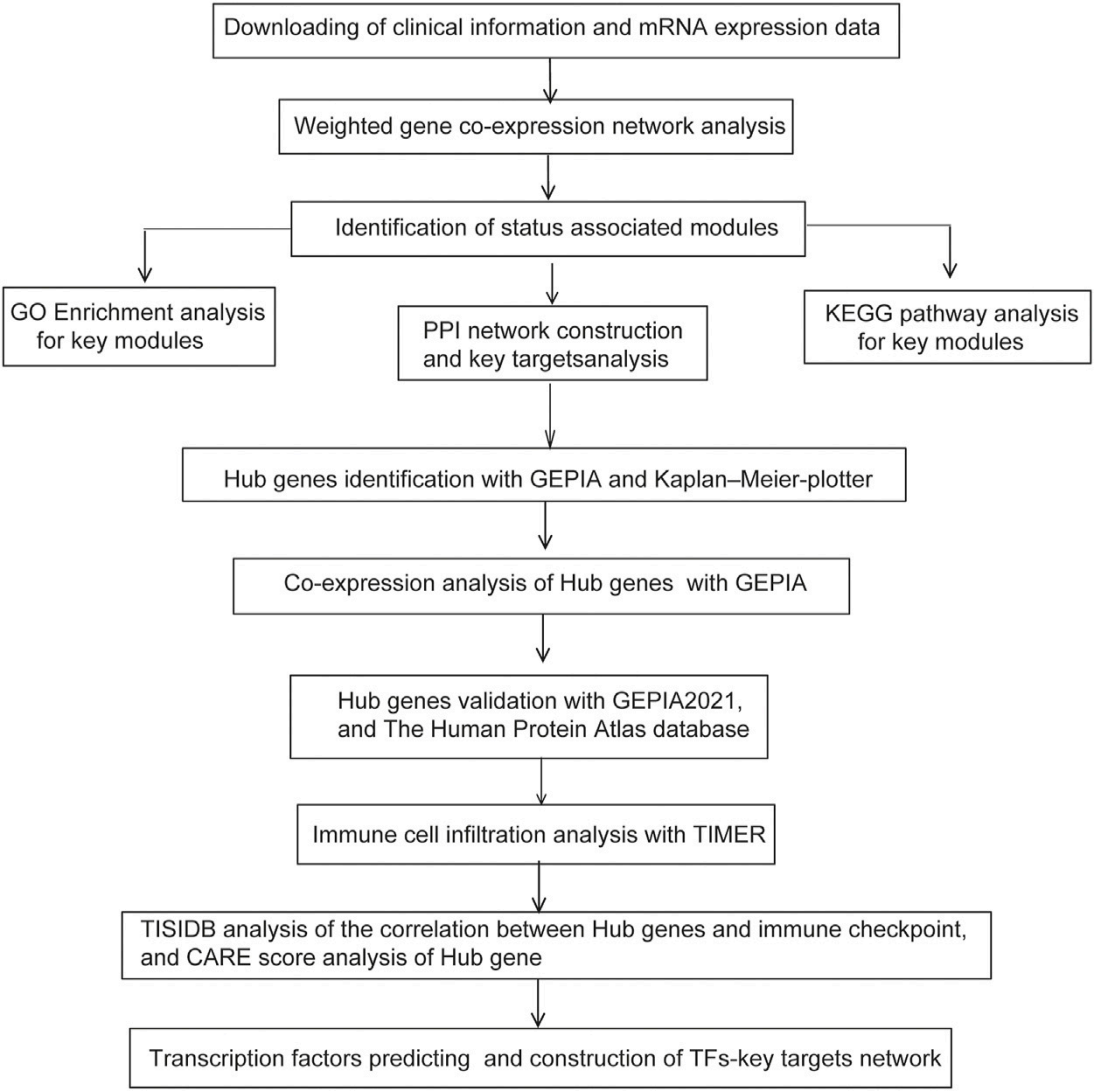
Experimental design and workflow of our study.

The general pipeline of the data analysis protocol was depicted in Figure 1. In general, GEO dataset of GSE102484 containing gene expression profiling data of 683 BRCA samples was analyzed using the R package WGCNA. The clinicopathological characteristics of tumor grade, T stage, N stage and metastasis were denoted. Remarkably, an 18-gene classifier, which could be applied to estimate the risks of local/regional recurrence (LRR) and distant metastasis in BRCA patients after mastectomy (Cheng et al., 2006; Cheng et al., 2017), was also denoted. After screening by MADs arranged from large to small, the expression of the top 25% genes (5,044 genes) with the greatest differences in samples were analyzed by WGCNA.

**FIGURE 2:**
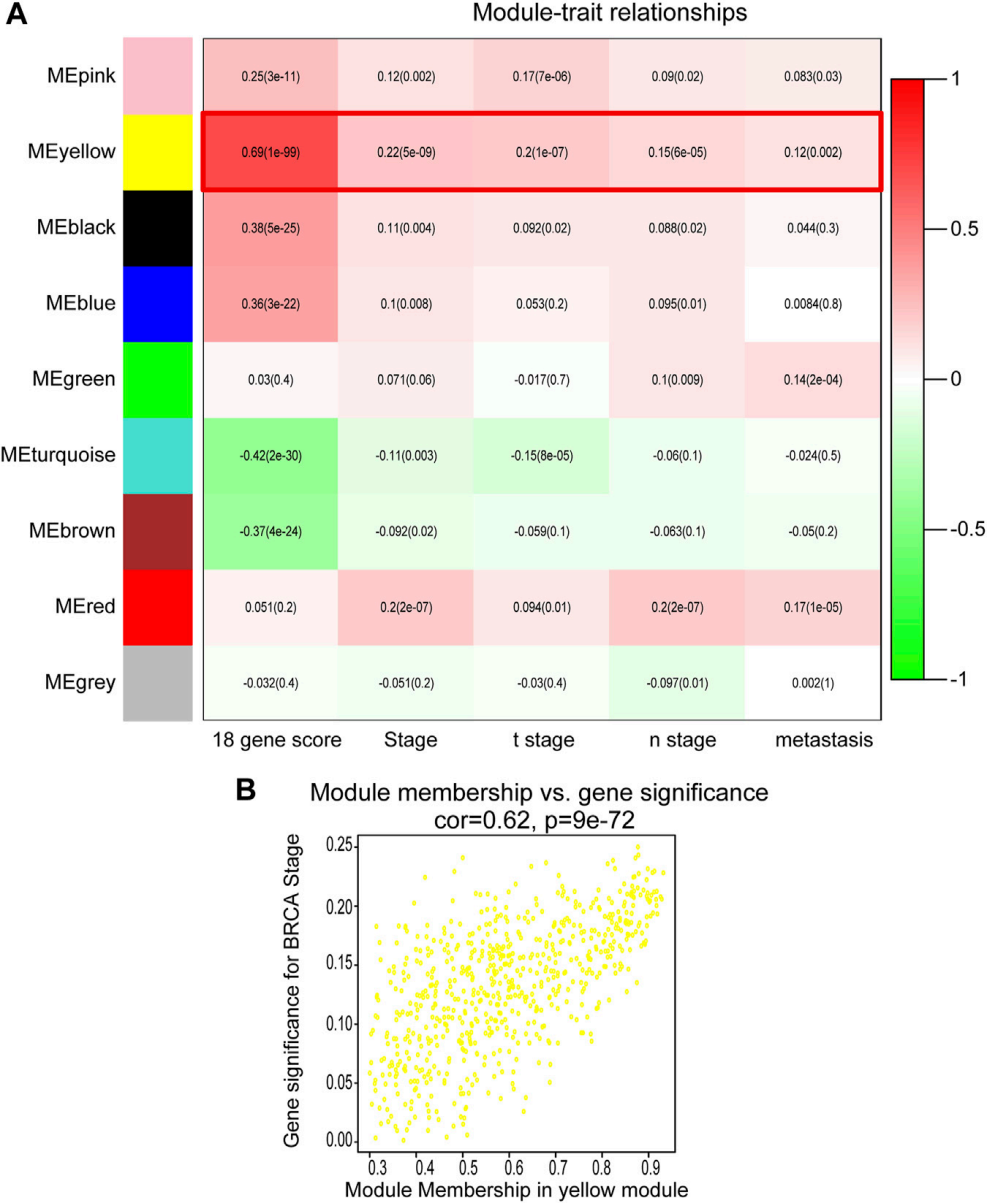
Identification of modules correlated with the clinical traits of BRCA. **(A)** Heatmap to show the correlation between modules and clinical features of BRCA. *p*-values are shown in brackets. **(B)** Scatter plot analysis to show the association between Module membership in the yellow module and gene significance for BRCA stage.

The DEGs with similar expression patterns were then grouped into different modules by average linkage clustering. As a result, nine modules were finally identified by merging similar modules when the MedissThres was set at 0.25 (Figure 2A). By calculating the correlation between eigengenes and the clinical traits, the yellow module was found to be more related to BRCA progression and metastasis as others. Scatter plot in Figure 2B further showed a significantly positive correlation between members of yellow module and gene significance for BRCA stage. Therefore, the yellow module was selected as the key module correlated with BRCA progression for follow-up analyses.

### Enrichment Analysis of the Yellow Module

**FIGURE 3:**
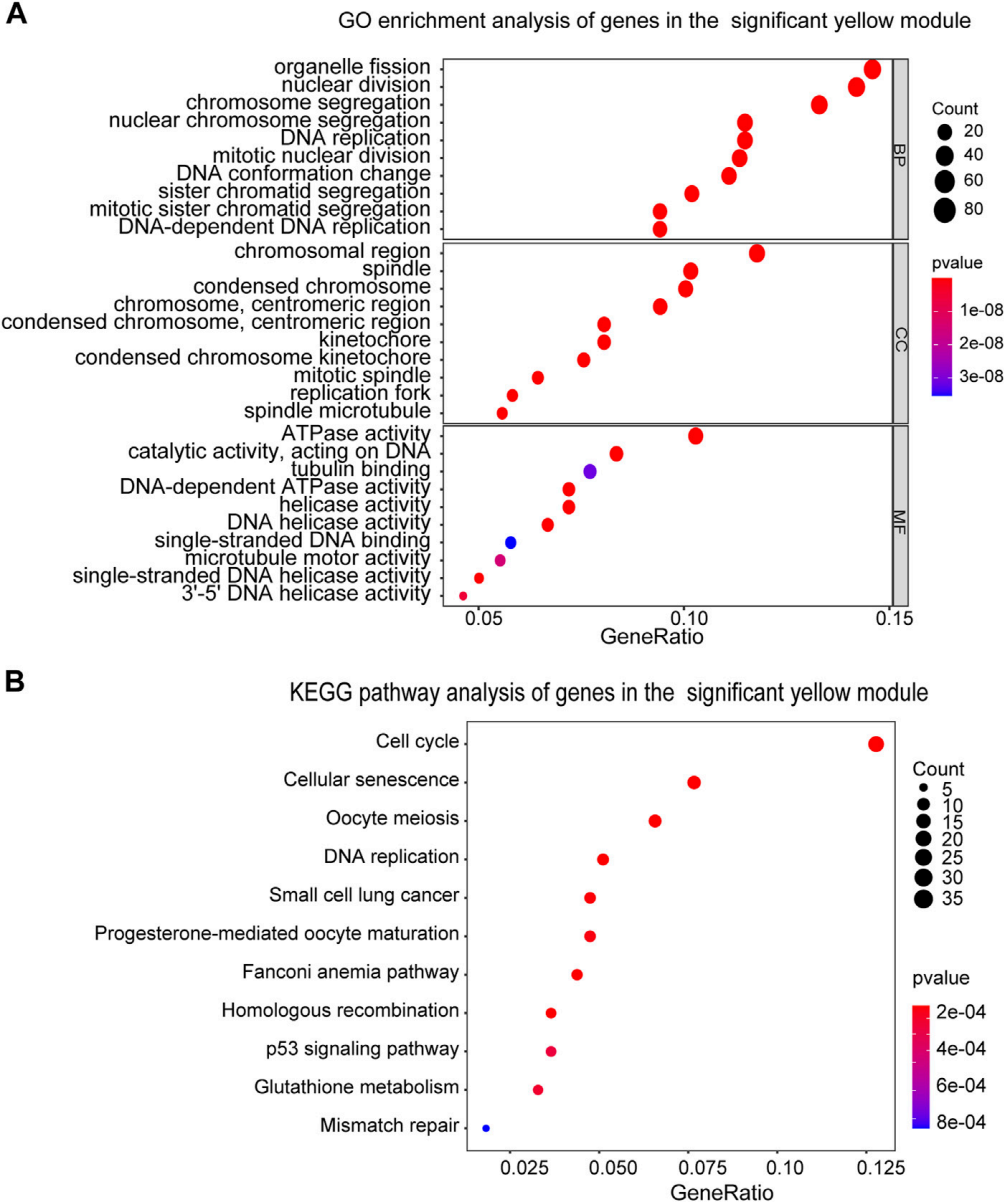
Functional enrichment analysis of genes in the yellow module. **(A)** GO enrichment analysis of the genes in the yellow module. BP, biological process, CC, cellular component, MF, molecular function. **(B)** KEGG pathway enrichment analysis of the genes in the yellow module. The sizes of the dots represent the number of genes in each term.

We next performed the GO and KEGG pathway analyses for the DEGs in the yellow module to illustrate their biological functions. In terms of GO analysis, Biological Process (BP) analysis led to the enrichment of these genes into the processes of organelle fission and nuclear division; while outcomes of Cellular Component (CC) and Molecular Function (MF) analyses resulted in the enrichment of these genes towards chromosomal region and ATPase activity (Figure 3A). Furthermore, KEGG pathway analysis result indicated that DEGs in the yellow module mainly exert their functions through cell cycle and cellular senescence pathways (Figure 3B).

### PPI Network Construction and Hub Genes Screening

**FIGURE 4:**
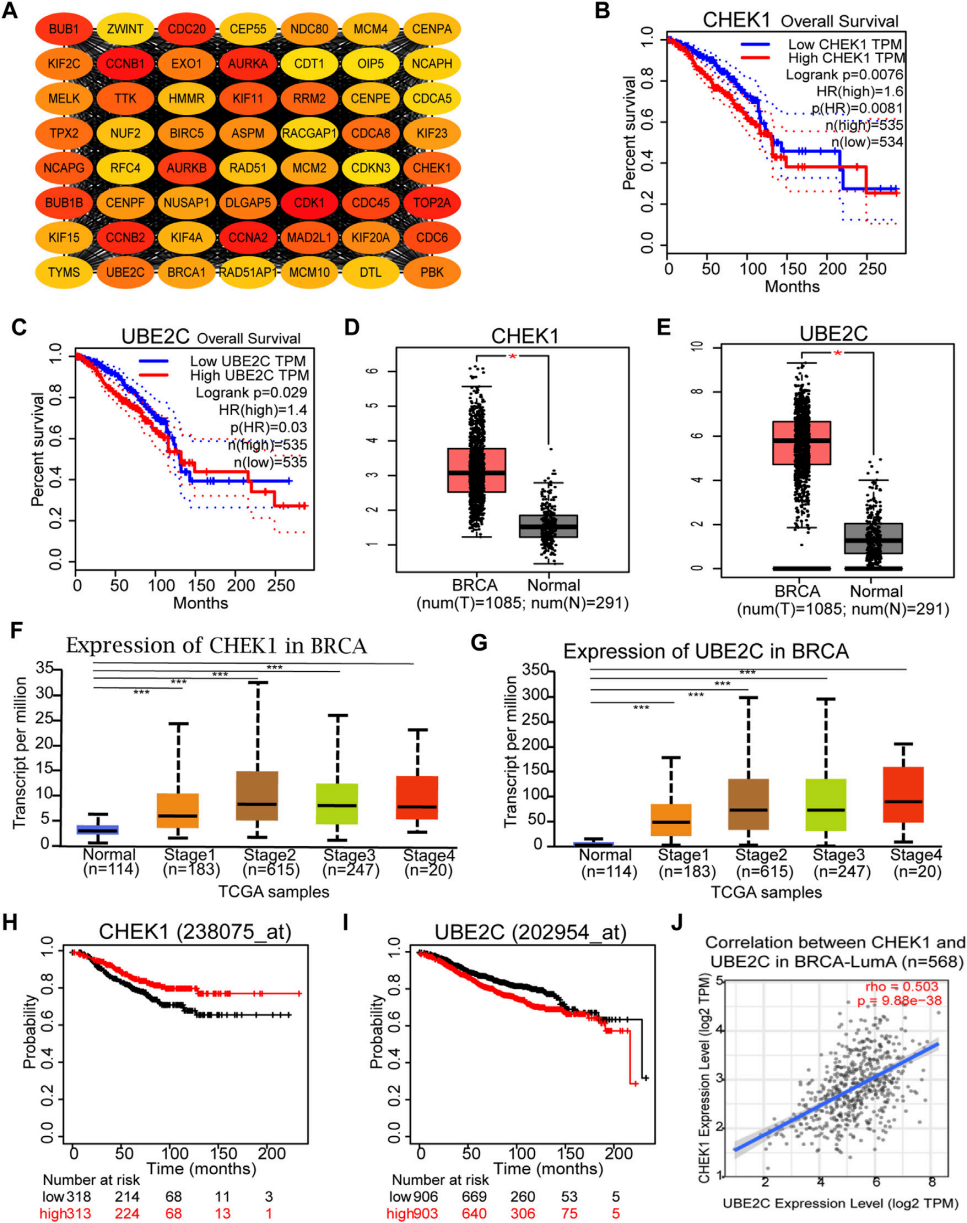
Identification and validation of Hub genes in BRCA. **(A)** Identification of the top 10% key targets from PPI network in the yellow module. **(B,C)** Overall survival of the two hub gene candidates in BRCA based on the Gene Expression Profiling Interactive Analysis (GEPIA) database. **(D,E)** Graphs showing the mRNA levels of two hub gene candidates in BRCA tissues compared with normal controls from GEPIA database. *p < 0.01 was considered as statistically significant. **(F,G)** Expression of CHEK1 and UBE2C in BRCA based on individual cancer stages. **(H,I)** Overall survival of the two hub genes in luminal-A subtype of BRCA based on Kaplan Meier-plotter. **(J)** Correlation Analysis of the two hub genes in luminal-A subtype of BRCA based on the TIMER2.0 database.

With the assistance of STRING database and Cytoscape software, a total of 664 DEGs in the yellow module were mapped into the PPI network, including 559 nodes and 12,050 edges. Subsequently, the top 10% key targets (56 genes) within PPI network were selected using cytoHubba plug-in in Cytoscape software based on rank of degree (Figure 4A). To shorten the scope and reconfirm our observations, both survival and differential expression analyses were conducted using the GEPIA web server. For all 56 hub gene candidates, only CHEK1 and UBE2C were found to be significantly correlated with BRCA patient survival and differentially expressed between cancer and normal specimens. To be specific, both genes were positively associated with poor patient survival rate (Figures 4B,C) and expressed higher in BRCA samples (Figures 4D,E). In addition, results of UALCAN analysis (Chandrashekar et al., 2022) suggested that elevated CHEK1 or UBE2C expression could be observed in BRCA tumors at the late stages (Figures 4F,G). To investigate the relationship between expression of the two selected candidates and BRCA subtypes, survival analysis was next performed using the survival information of each BRCA subtype in Kaplan–Meier plotter. As a result, elevated expression of both genes was only observed to be significantly associated with poor outcome of patients diagnosed with luminal-A type of BRCA (Figures 4H,I). Interestingly, expressions of CHEK1 and UBE2C were positively correlated with each other in luminal A breast cancer subtype (Figure 4J), suggesting the existence of a potential co-regulatory network for both genes. Collectively, our data suggested that CHEK1 and UBE2C could be served as hub genes associated with BRCA progression, especially for the luminal-A subtype.

### Expression of Hub Genes in Different Cell Populations of Breast Cancer

**FIGURE 5:**
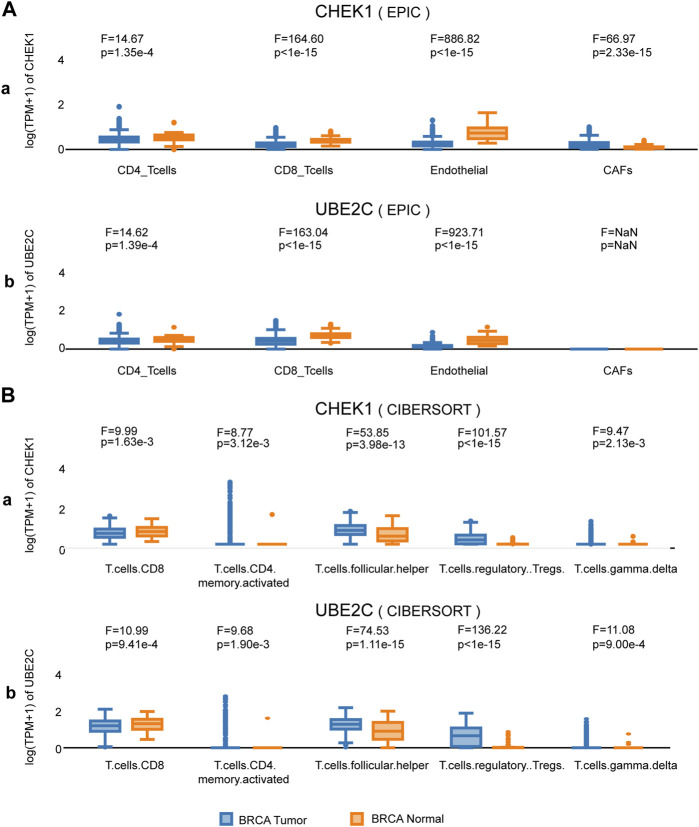
Expression analysis of the two hub genes at cell-type level in BRCA. **(A)** Expression of the two hub genes in various clusters of human immune cells from BRCA by deconvolution tool EPIC. **(B)** Expression of the two hub genes in various clusters of human immune cells from BRCA by deconvolution tool CIBERSORT.

We next sought to investigate the expression of CHEK1 and UBE2C in BRCA at cell type level with the help of GEPIA 2021 (Li et al., 2021). By using the deconvolution tool of EPIC, we observed significant upregulation of both hub genes in CD4^+^ T cells, CD8^+^ T cells and endothelial cells (Figure 5A). These findings were further confirmed through the deconvolution tool of CIBERSORT (Figure 5B). Meanwhile, downregulation of Follicular Helper, Tregs and Gamma delta T cells were also found in both hub genes (Figure 5B), indicating that CHEK1 and UBE2C were coordinately expressed in various cell populations of BRCA.

### Immune Cell Infiltration Analysis of the Hub Genes

**FIGURE 6:**
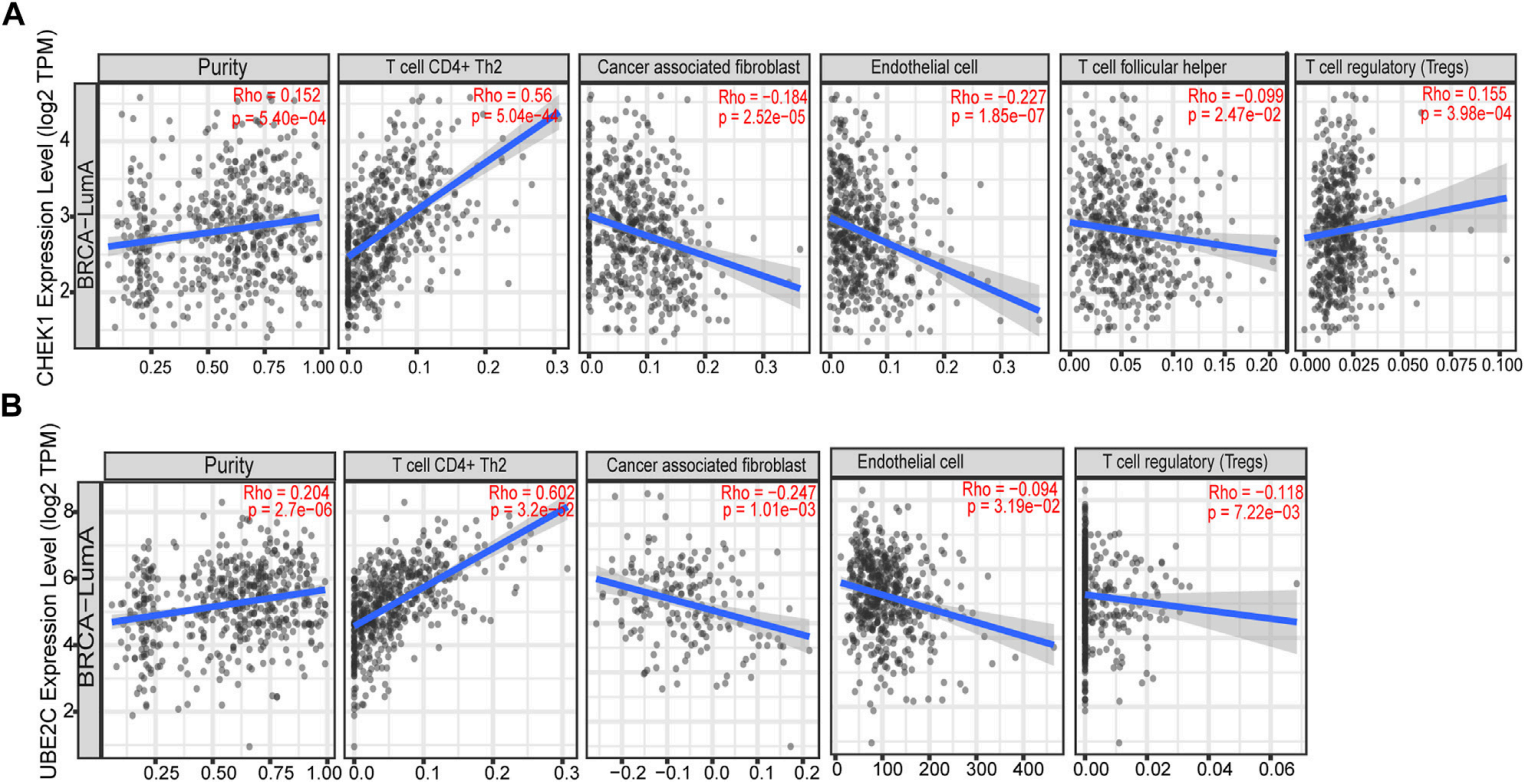
Correlation of the expression of two hub genes with immune infiltration level in BRCA based on TIMER. **(A)** Correlation of the expression of CHEK1 with immune infiltration level in luminal-A subtype of BRCA. **(B)** Correlation of the expression of UBE2C with immune infiltration level in luminal-A subtype of BRCA.

To determine whether any correlation is existed between tumor infiltration with immune cells and expression of hub genes identified in this study, the tumor infiltration across multiple immune BRCA cells was analyzed by TIMER along with other tools. As presented in Figures 6A,B, strong positive correlation was observed between expression of two hub genes and infiltrating levels of CD4^+^ T cells, while weak negative correlation could be found between expression of two hub genes and infiltrating levels of cancer associated fibroblast or endothelial cells.

**FIGURE 7:**
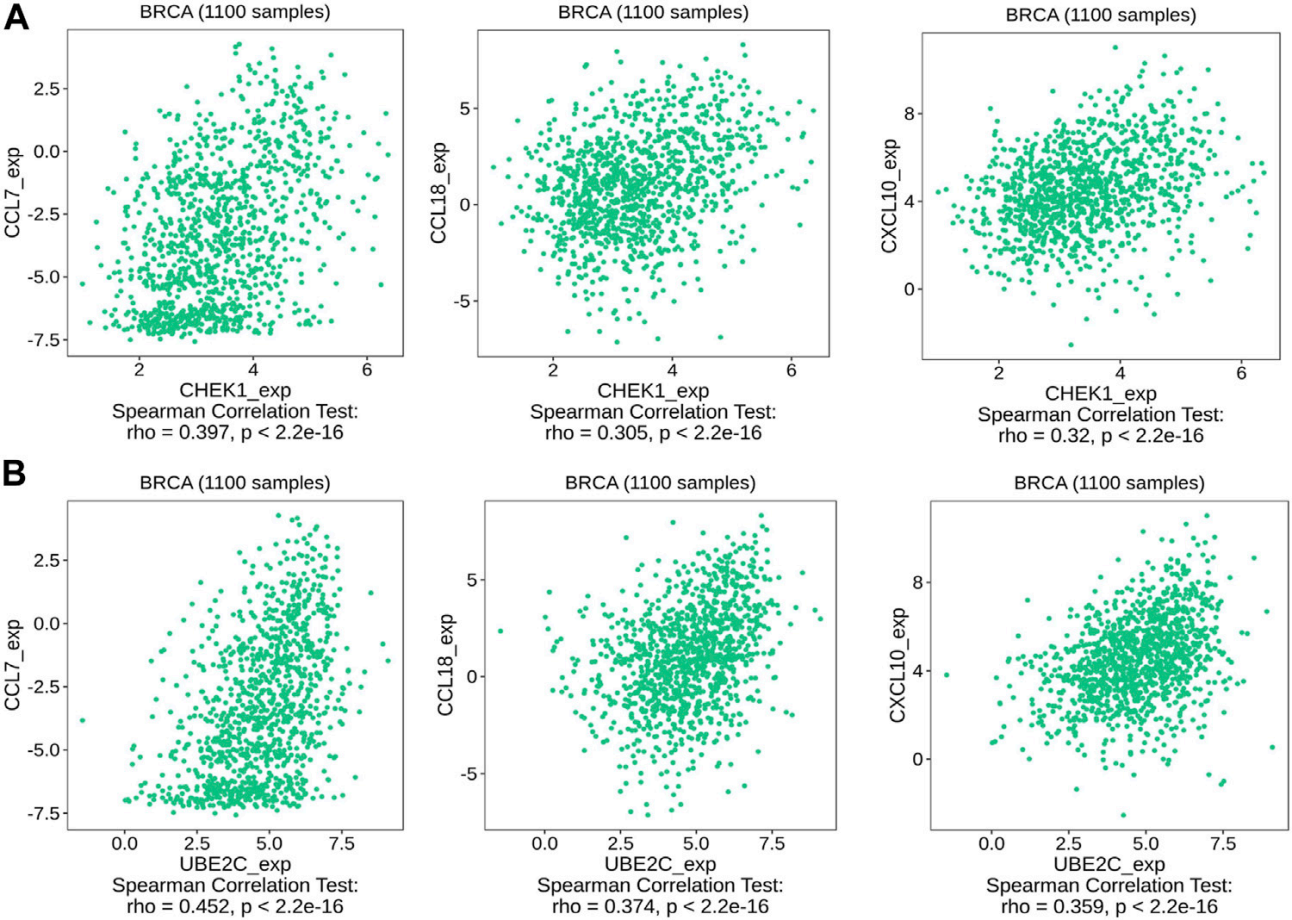
Relations between the expression of the two hub genes and chemokines of CCL7, CCL18, CXCL10. **(A)** Relations between expression of CHEK1 and three chemokines in BRCA. **(B)** Relations between expression of UBE2C and three chemokines in BRCA.

**FIGURE 8:**
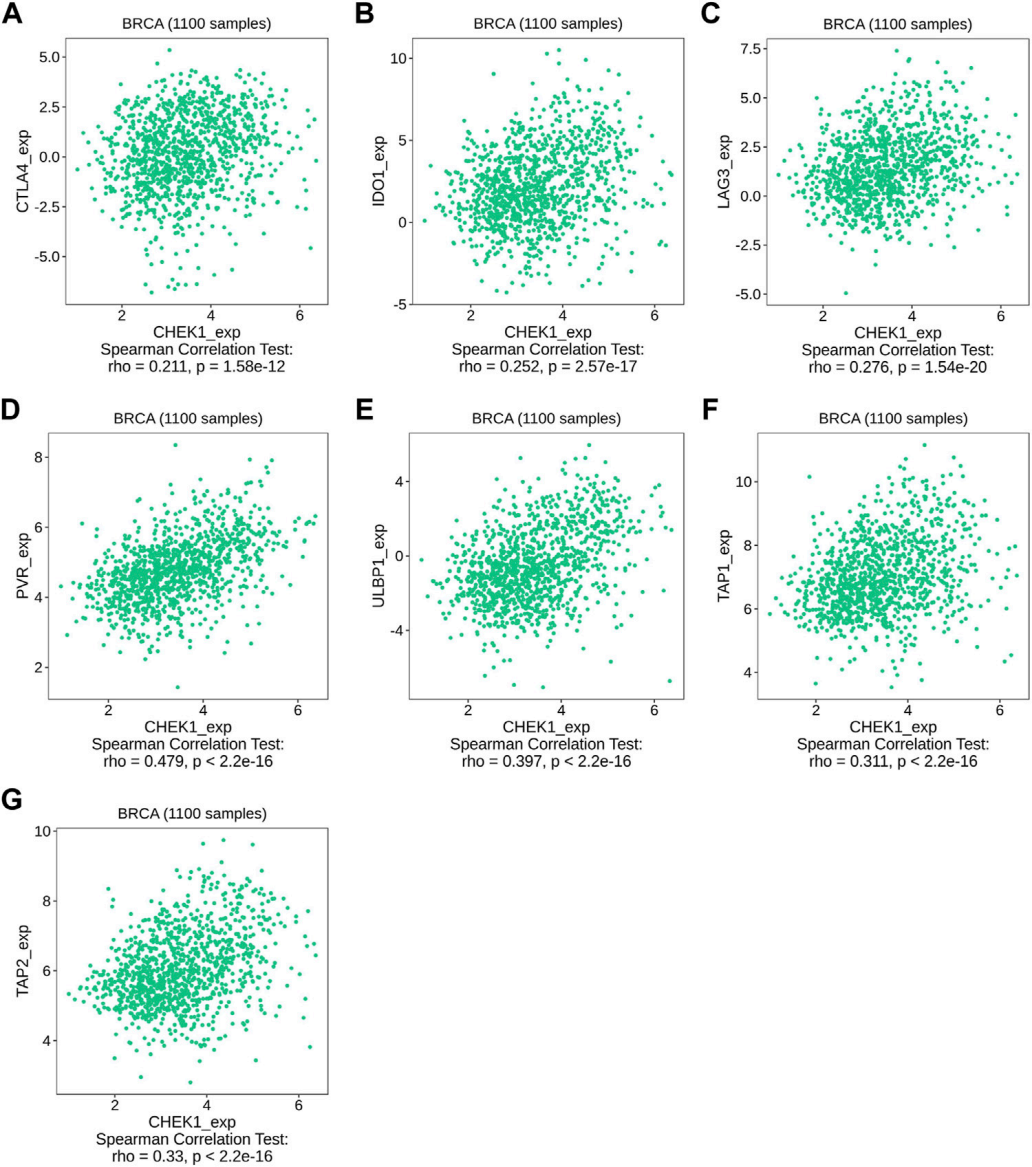
Relations between the expression of CHEK1 and immune checkpoints in BRCA. **(A–C)** Relations between three immunoinhibitors (CTLA4, IDO1, LAG3) and expression of CHEK1. **(D,E)** Relations between two immunostimulators (PVR and ULBP1) and expression of CHEK1. **(F,G)** Relations between two MHC molecules (MHC_TAP1 and MHC_TAP2) and expression of CHEK1.

**FIGURE 9:**
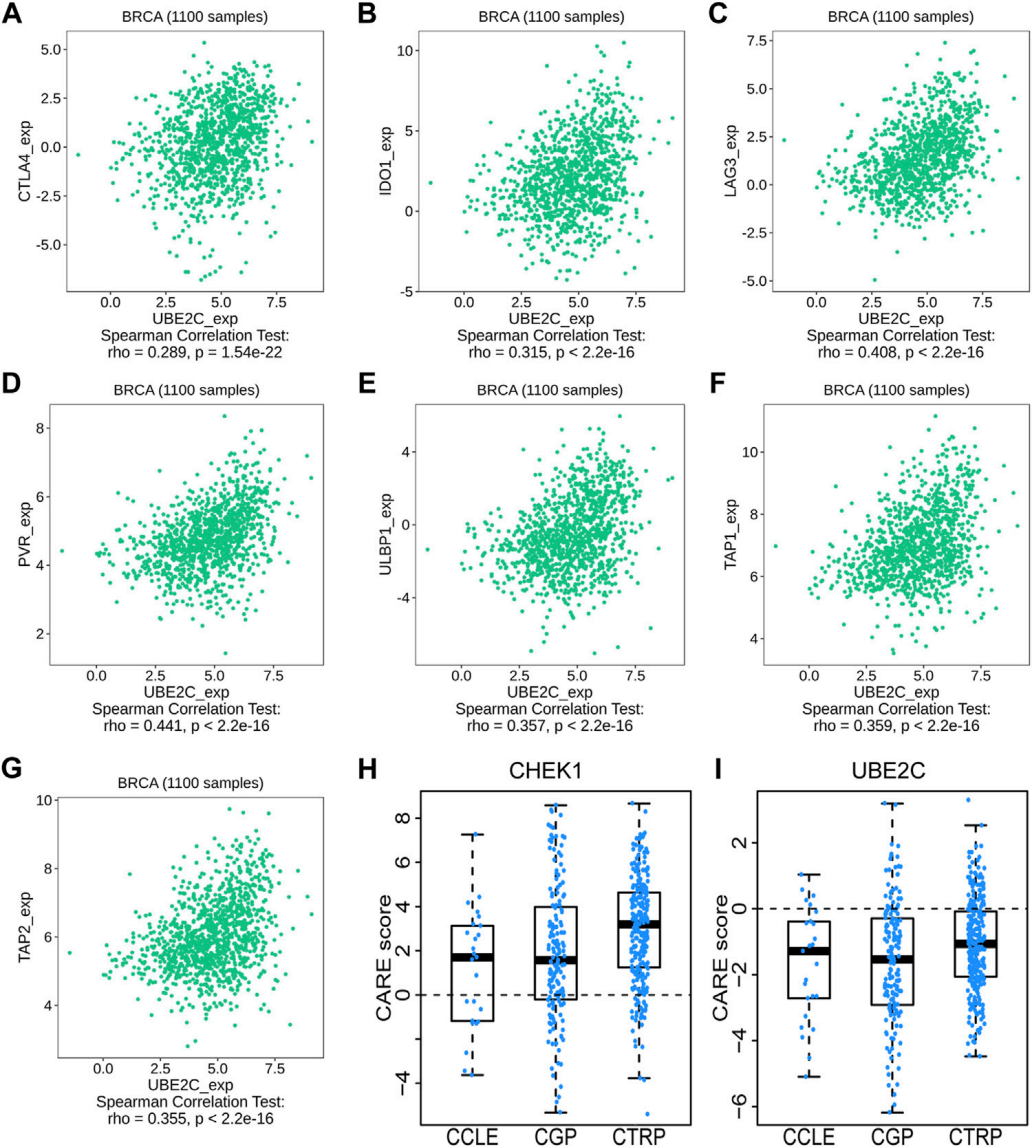
Relations between the expression of UBE2C and immune checkpoints in BRCA. **(A–C)** Relations between three immunoinhibitors (CTLA4, IDO1, LAG3) and expression of UBE2C. **(D,E)** Relations between two immunostimulators (PVR and ULBP1) and expression of UBE2C. **(F,G)** Relations between two MHC molecules (MHC_TAP1 and MHC_TAP2) and expression of UBE2C. **(H,I)** The CARE score of CHEK1 **(H)** and UBE2C **(I)** on CCLE, CGP, CTRP dataset.

We then used TISIDB to detect the association between hub genes and immune checkpoints. Intriguingly, both CHEK1 and UBE2C were found to be positively correlated with the expression of a series of immune checkpoints, including chemokines of CCL7, CCL18 and CXCL10; immunoinhibitors of CTLA4, IDO1 and LAG3; immunostimulators of PVR and ULBP1 and MHC molecules of TAP1 and TAP2 (Figures 7, 8, 9A–G). To gain more insights of these observations, the CARE approach was next applied to evaluate how hub genes interact with other genes to affect drug efficacy. As a result, CHEK1 showed significantly positive CARE scores for multiple compounds screened in all three cohorts (Figure 9H), suggesting that high expression of CHEK1 is associated with better response to immunotherapy. In sharp comparison, significantly negative CARE scores were observed for UBE2C (Figure 9I), indicating that expression of UBE2C may promote drug resistance towards many targeted therapies.

### The Transcriptional Regulatory Network of Hub Genes and Transcription Factors

**FIGURE 10:**
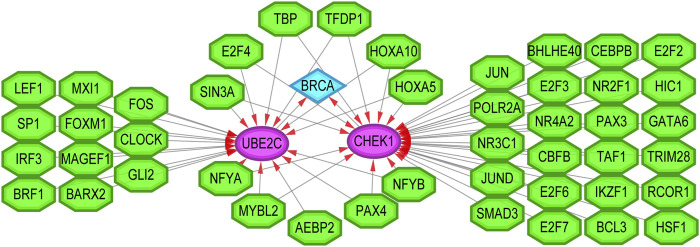
The transcriptional regulatory network of TFs and the two hub genes. TFs: transcription factors. Green hexagon nodes represented TFs, pink circular nodes represented TFs regulated hub genes, and light blue diamond node represented the BRCA. Their interactions were represented by arrows. The number of arrows in the networks demonstrated the contribution of one TF to the hub genes. The higher the degree the more central the nodes were within the network.

Finally, we sought to establish the transcriptional regulatory network between two hub genes and TFs by the plugin iRegulon of Cytoscape. As revealed in Figure 10, a total of 45 TFs were identified to be involved in the regulation of CHEK1 and UBE2C. Notably, 11 of them were characterized as the upstream regulators of both hub genes. Future studies focusing on these TFs may shed light on the understanding of the co-activated expression pattern of CHEK1 and UBE2C during the progression of BRCA.

## Discussion

It is generally accepted that effective driver genes could play essential roles during the tumorigenesis and development of cancer (Dawson et al., 2013; Martínez-Jiménez et al., 2020). However, till now, only a limited number of gene drivers, such as ER (Jensen and DeSombre, 1973), HER2(Slamon et al., 1987) and EZH2 (Adibfar et al., 2021; Yi et al., 2021; Yi et al., 2022), have been fully validated as targetable oncogenic drivers of BRCA. In the current study, CHEK1 and UBE2C were newly identified as hub genes and potential therapeutic targets of BRCA with the help of WCGNA, TIMER and other powerful bioinformatic tools.

As a conserved serine/threonine kinase and component of several cell cycle checkpoints, cell cycle checkpoint kinase 1 (CHEK1) is required for replication fork stabilization and DNA damage response (Dai and Grant, 2010; Blasius et al., 2011). Genomic instability induced by impairment of cell cycle checkpoints is widely recognized as a hallmark of cancer. Thus, CHEK1 could be considered as an attractive target for cancer-specific therapy (Rundle et al. et al., 2017). Currently, multiple CHEK1 inhibitors have been commercially developed and showed promising results in pre-clinical studies for cancer types like lymphoma (Walton et al., 2016) and neuroblastoma (Walton et al., 2012). Based on our findings in the current study, it is anticipated that targeted therapy using CHEK1 inhibitors may also lead to optimal outcomes for BRCA patients.

Similar to CHEK1, Ubiquitin-conjugating enzyme 2C (UBE2C) also participates in cell cycle progression and checkpoint control by targeting abnormal or short-lived proteins for degradation (Hao et al., 2012). Overexpression of UBE2C has been reported in various human tumors, which leads to chromosomes mis-segregation and uncontrolled cell cycle process in cancer (Chen et al., 2010; van Ree et al., 2010; Zhao et al., 2013). In terms of BRCA, tumor suppressor BRCA1 was identified as a negative regulator of UBE2C which could thereby promote the sensitivity of BRCA cells to doxorubicin (Qin et al., 2017). In addition, microRNA-196a has been validated to upregulate UBE2C post-transcriptionally and thus promotes cell proliferation in BRCA (Han et al., 2015). In accordance with the previous studies, our data reconfirm the significance of UBE2C during carcinogenesis of BRCA and further prove that UBE2C might regulate the development of BRCA by affecting the T cell functions. However, further experiments are needed to unveil the underlying mechanism by which these two hub genes modulate tumor infiltration.

Through regulatory network analysis, we identified 11 TFs (SIN3A, E2F4, TBP, TFDP1, HOXA5, HOXA10, NFYA, MYBL2, AEBP2, PAX4 and NFYB) that could target both CHEK1 and UBE2C in BRCA. Interestingly, most of them exhibited an elevated expression pattern in BRCA based on The Cancer Genome Atlas (TCGA) database (Hutter and Zenklusen, 2018). Thus, it is not surprising that upregulation of these TFs may lead to the co-activation of CHEK1 and UBE2C, which subsequently facilitates the progression of BRCA.

In summary, through integrated bioinformatics analysis, our present study identified CHEK1 and UBE2C as potential prognostic and therapeutic targets in BRCA. Moreover, both candidates may be served as effective biomarkers to evaluate the immune status of BRCA patients and predict the effectiveness of immunotherapy before treatment.

## Data Availability

The original contributions presented in the study are included in the article/Supplementary Material, further inquiries can be directed to the corresponding authors.
